# Supplementary material for: Effects of breeding history and crop management on the root architecture of wheat
Source: Plant Soil. 2020 Jun 20;452(1):587–600. doi: 10.1007/s11104-020-04585-2 (PMC7371663; doi:10.1007/s11104-020-04585-2)
Supplement: Supplementary file 1 — (DOCX 23 kb) [file 11104_2020_4585_MOESM1_ESM.docx]

**Supplementary Material**

**Supplementary Table 1.** Origins and release dates of winter wheat cultivars as part of the WHEALBI panel. Dates of use for some genotypes are unavailable. Database ID with prefixes ‘PI’ are from the USDA GRIN database (https://npgsweb.ars-grin.gov/); ‘GRU’ and ‘WAT’ from the Germplasm Resource Unit and Watkins Collection (https://www.seedstor.ac.uk); ‘WW’ are WHEALBI accessions (http://www.whealbi.eu/); ‘URGI’ from GnpIS (https://urgi.versailles.inra.fr/gnpis/).

| Genotype name | WHEALBI ID | Database ID | Collection group | Country of origin | Release date or approximate date of first use |
| --- | --- | --- | --- | --- | --- |
| Alchemy | WW-048 | URGI-29921 | Modern variety | UK | 2006 |
| Bankuti 1201 | WW-089 | PI-232943 | Landrace | Hungary | 1931 |
| Cappelle Desprez |  | GRU-W0385 | UK historic | France/UK | 1946 |
| Hereward | WW-051 | GRU-W9448 | Modern variety | UK | 1991 |
| JB Diego |  | URGI-36542 | Modern variety | UK | 2002 |
| KWS Santiago | WW-030 | GRU-W10338 | Modern variety | UK | 2011 |
| Maris Wigeon |  | GRU-W4114 | UK historic | UK | 1964 |
| Milns N 59 |  | GRU-W0505 | UK historic | UK | 1951 |
| MV Kolo | WW-026 | URGI-36434 | Modern variety | Hungary | 2006 |
| Ostka Skomoroska |  | PI-285873 | Landrace | Poland | 1920 |
| Red Lammas |  | GRU-W0987 | UK historic | UK | ~1740 |
| Red Standard |  | GRU-W5623 | UK historic | UK | ~1905 |
| Red Stettin 13 |  | GRU-W1091 | UK historic | Ireland | 1850 |
| Robigus | WW-053 | GRU-W9999 | Modern variety | UK | 2003 |
| Samanta 117 |  | PI-278425 | Landrace | Romania | <1962 |
| Soissons | WW-054 | GRU-W9465 | Modern variety | UK | 1995 |
| Steadfast |  | GRU-W0513 | UK historic | UK | 1942 |
| Tiepolo | WW-021 | URGI-38984 | Modern variety | Italy | 2009 |
| WW 502 (China Sh12) | WW-502 | WAT1190440 | Landrace | China | - |
| WW 512 (Italy W8457) | WW-512 | WAT1190816 | Landrace | Italy | - |

**Supplementary Table 2.** Origins and release dates of the 16 wheat varieties that represent the founders of the NIAB Diverse MAGIC population.

| **Name** | **Country of origin** | **Adaptation** | **Release date** |
| --- | --- | --- | --- |
| Banco | Sweden | Winter | 1956 |
| Bersee | UK/France | Winter | 1951 |
| Brigadier | UK | Winter | 1993 |
| Copain | France | Winter | 1980 |
| Cordiale | UK | Winter | 2004 |
| Flamingo | NL/DK | Winter | 1960 |
| Gladiator | UK | Winter | 2004 |
| Holdfast | UK | Winter | 1935 |
| Kloka | Germany | Facultative | 1965 |
| Maris Fundin | UK | Winter | 1975 |
| Robigus | UK | Winter | 2003 |
| Slejpner | Denmark/Sweden | Winter | 1986 |
| Soissons | France | Winter | 1995 |
| Spark | UK | Winter | 1993 |
| Steadfast | UK | Winter | 1942 |
| Stetson | UK | Winter | 1983 |

**Supplementary Table 3.** Soil chemistry measurements at the Sonning site in both years.

|  | **Year 1** | **Year 2** |
| --- | --- | --- |
| pH | 6.2 | 6.0 |
| NO_3_ -N (mg kg-1) | 4.08 | 0.98 |
| P (mg l-1) | 30.6 | 40.4 |
| P Index | 3 | 3 |
| K (mg l-1) | 59.6 | 196.0 |
| K Index | 0 | 2+ |
| Mg (mg l-1) | 37.6 | 68.0 |
| Mg Index | 1 | 2 |
| Organic matter (%) | 2.6 | 2.4 |

**Supplementary Table 4**. Raw data per plot used for analysis from both the Duxford and Sonning trials sites.
